# A case of probable Vogt–Koyanagi–Harada disease in a 3-year-old girl

**DOI:** 10.1186/s12886-019-1192-0

**Published:** 2019-08-13

**Authors:** Atsuko Katsuyama, Sentaro Kusuhara, Hiroyuki Awano, Hiroaki Nagase, Wataru Matsumiya, Makoto Nakamura

**Affiliations:** 1 Division of Ophthalmology, Department of Surgery, Kobe University Graduate School of Medicine, 7-5-1 Kusunoki-cho, Chuo-ku, Kobe, 650-0017 Japan; 2 Department of Pediatrics, Kobe University Graduate School of medicine, Kobe, Japan

**Keywords:** Vogt–Koyanagi–Harada disease, Child, Uveitis, Corticosteroid therapy

## Abstract

**Background:**

Vogt–Koyanagi–Harada (VKH) disease is a T-cell-mediated autoimmune disorder characterized by bilateral granulomatous panuveitis with various systemic manifestations. Although VKH disease rarely occurs in the pediatric population, the clinical course tends to be aggressive, and the visual prognosis is worse than that in adult patients due to severe ocular complications secondary to recurrent inflammation.

**Case presentation:**

A 3-year-old girl with probable VKH was referred to Kobe University Hospital. She had severe bilateral panuveitis with posterior synechiae of the iris, marked optic disk swelling, and serous retinal detachment in both eyes, and her best corrected visual acuities (BCVAs) were 20/200 OD and 20/125 OS. A third course of therapy was administered because serous retinal detachment remained after two courses of therapy. She was treated with three courses of high-dose intravenous corticosteroid therapy, followed by slow tapering of oral corticosteroids. Her BCVAs recovered to 20/16 OU, and relapse of ocular inflammation and side effect of treatment were not observed during the 1.5-year follow-up period.

**Conclusions:**

We experienced a pediatric patient with probable VKH disease who was treated with three courses of high-dose intravenous corticosteroid therapy. With the favorable clinical course in our patient, initial treatment with repeated high-dose intravenous corticosteroid therapy might be beneficial in pediatric VKH disease.

## Background

Vogt–Koyanagi–Harada (VKH) disease is an autoimmune disorder driven by melanocyte antigen-reactive T-cells characterized by bilateral granulomatous panuveitis with systemic manifestations, such as tinnitus, vertigo, and meningismus [1]. VKH rarely occurs in the pediatric population. However, the course of the disease in children is reported to be more aggressive than that in adults [2, 3]. Abu El-Asrar and associates reviewed 23 cases (46 eyes) of VKH disease in children. They found that visual acuity of 20/40 or better had been achieved in 82.6% of eyes. They extracted the factor associated with poor visual prognosis and development of complications, such as cataract and glaucoma, which is significantly associated with recurrence of inflammation [4]. Here we present a case of probable VKH disease in a 3-year-old girl with several risk factors for poor visual prognosis before treatment and who was successfully treated with intensive corticosteroid therapy.

## Case presentation

A 3-year-old girl with a 1-month history of sustained conjunctival injections of both eyes was presented to a local eye clinic. After failure of treatment with an anti-allergic eye drop, she was prescribed with eye drops (0.5% levofloxacin, 0.1% betamethasone, and 0.5% tropicamide/0.5% phenylephrine) and was referred to the Department of Ophthalmology at Kobe University Hospital for the treatment of bilateral panuveitis.

**Fig. 1:**
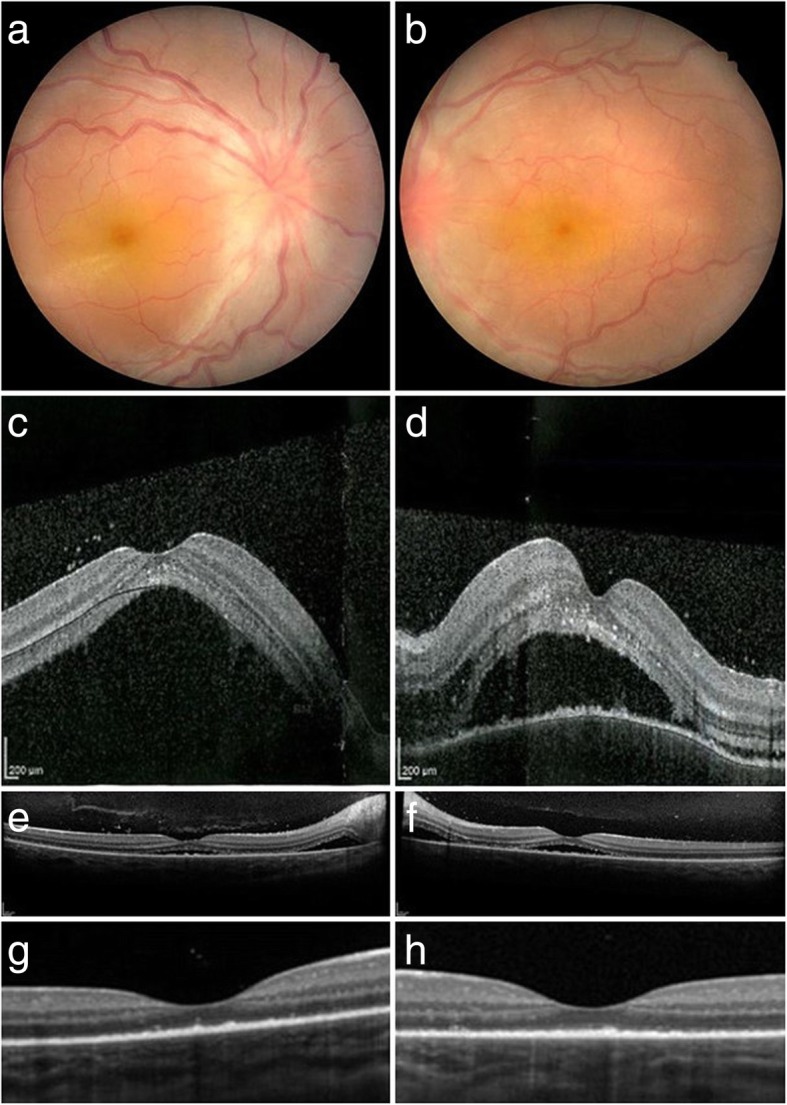
Color fundus photographs at presentation and changes of OCT images after steroid therapy (**a**, **b**). Fundus images at presentation. Optic disk swelling and tortuous retinal vessels are observed in both eyes (**c**, **d**). OCT images at presentation. Bilateral marked serous retinal detachment is shown (**e**, **f**). OCT images after two courses of high-dose intravenous corticosteroid therapy. Serous retinal detachment is still present (**g**, **h**). OCT images after three courses of high-dose intravenous corticosteroid therapy. No subretinal fluid is seen, but the integrity of EZ is lost in both eyes

She had an unremarkable medical and family history and denied any other ocular or systemic symptom including ocular pain and headache. We confirmed that she had no history of penetrating trauma or surgery preceding this initial onset of uveitis. On examination, best corrected visual acuities (BCVAs) were 20/200 OD and 20/125 OS, and the intraocular pressure was 7 mmHg in the left eye (a reliable value was not obtained in the right eye). Slit-lamp biomicroscopy revealed posterior synechiae of the iris in both eyes. Detailed information on the anterior chamber was not obtained due to poor patient cooperation (she looked to be sensitive to light). Funduscopy revealed bilateral optic disk swelling and tortuous retinal vessels, and optical coherence tomography (OCT) revealed serous retinal detachment with choroidal thickening in both eyes (Fig. 1, a–d). The results of blood test, electrocardiography, and chest X-ray were all normal. Fluorescence angiography (FA), indocyanine green angiography (ICGA), and cerebrospinal fluid (CSF) examination were not performed, considering her age and cooperation and due to the parents’ will. As she did not exhibit any neurological or auditory finding on presentation, she was diagnosed with probable VKH disease based on the international criteria [5]. A pediatrician consulted her, and she was admitted to the hospital on the day of the referral.

**Fig. 2:**
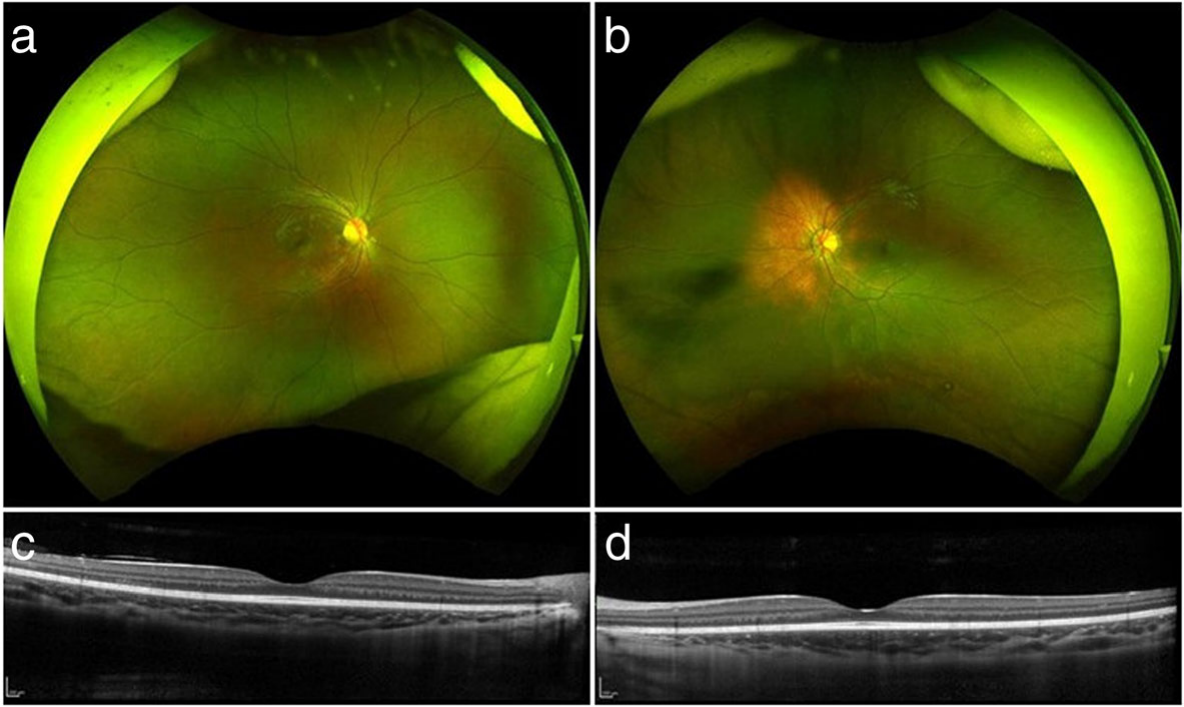
Ultra-widefield color fundus photographs and OCT images 1 year after presentation (**a**, **b**). Ultra-widefield color fundus imaging shows normal results in the right eye and a limited depigmented area around the optic disk in the left eye (**c**, **d**). OCT imaging demonstrates a normal macular morphology with restored EZ in both eyes

On the following day, high-dose intravenous corticosteroid therapy (methylprednisolone 30 mg/kg/day for three consecutive days) was started. She received three courses of high-dose intravenous corticosteroid therapy given at a 1-week interval because subretinal fluid remained on OCT after two courses of therapy (Fig. 1, e and f). Subretinal fluid completely disappeared after the third intravenous corticosteroid therapy, but BCVAs improved to 20/50 OD and 20/40 OS with disorganized ellipsoid zone (EZ) on OCT (Fig. 1, g and h). Oral prednisolone was administered at a daily dose of 0.4 mg/kg, which gradually tapered off over the following 6 months. An experienced pediatrician had monitored the patient’s health and growth throughout the treatment period. One year after treatment, she recovered her vision (20/16 OU). Ultra-widefield fundus imaging revealed no apparent sunset glow fundus in the right eye and a limited depigmented area around the optic disk in the left eye. OCT revealed intact EZ in both eyes (Fig. 2). Neither relapse of ocular inflammation nor side effect of treatment such as cataract formation, intraocular pressure (IOP) rise above 20 mmHg and growth retardation was observed during the 18-month follow-up period (17-month follow-up period after the third course of high-dose intravenous corticosteroid therapy).

## Discussion and conclusions

We experienced a 3-year-old case of probable VKH disease who was successfully treated with intensive corticosteroid therapy. We made a diagnosis of probable VKH based on the international criteria [5]. If we had perfomed additional tests such as FA and CSF examination, the diagnosis might have changed from ‘probable’ to ‘incomplete’ or ‘complete’ VKH disease. However, the parents refused those tests and integumentary findings such as poliosis or vitilligo were not observed during the clinical course (probably due to successful treatment). Therefore, we had no choice but to treat the patient under the diagnois of probable VKH disease. The diseases that should be diffrentiated would be central serous chorioretinopathy and scleritis. The former can be excluded by the favorable response to corticosteroid therapy and the latter by the lack of ocular pain and the normal blood test results.

A retrospective case review of VKH disease reported that the ratio of final BCVA of 20/200 or worse in the pediatric group (61%) was significantly higher than that in the adult group (26%) because of aggressive inflammation and subsequent severe ocular complications [2]. In our patient, the ocular inflammation was so severe that three courses of high-dose intravenous corticosteroid therapy were required to resolve the serous retinal detachment. Before treatment, our patient had several prognostic factors associated with recurrent inflammation, including initial BCVA of ≤20/200, posterior synechiae of the iris at presentation, and an interval of > 2 weeks between symptoms and treatment [4]. Therefore, we presumed that complete resolution of inflammation by intensive corticosteroid therapy, followed by slow tapering of oral corticosteroids, would contribute to the prophylaxis of recurrent inflammation. Previous reports indicated that the clinical course of VKH disease occurring in patients younger than 5 years would be variable [2, 6–9]. Two patients treated with intensive corticosteroid (intravenous methylprednisolone, 30 mg/kg/day for 3 days, followed by oral prednisolone tapered from 1.5 to 2.0 mg/kg/day) had a favorable course [7, 8], whereas three patients treated with smaller amounts of corticosteroids had poor prognosis with BCVAs of no light perception of 20/200 [2, 9]. With this information, the recommendation of a pediatrician who had an enough experience to treat pediatric patients with high-dose intravenous corticosteroid therapy, and the presence of residual subretinal fluid after two courses of high-dose intravenous corticosteroid therapy, we decided to add the third course. Although we successfully treated our patient with multiple courses of high-dose intravenous corticosteroid therapy and no side effects were observed so far, a long-term follow-up should be needed. If recurrence of inflammation or corticosteroid-related side effects might occur, different treatment regimens using immunosuppressive agent or biologic agents should be considered [10].

In conclusion, the favorable clinical course in our patient suggests that initial treatment with repeated high-dose intravenous corticosteroid therapy might be beneficial in pediatric patients with VKH disease who have risk factors for recurrent inflammation.

## Data Availability

All data supporting these findings are contained within this manuscript.

## Abbreviations

BCVA: Best corrected visual acuity
EZ: Ellipsoid zone
OCT: Optical coherence tomography
VKH: Vogt–Koyanagi–Harada
